# Chronic Spontaneous Urticaria – the Saskatchewan experience and questionnaire survey

**DOI:** 10.1186/1710-1492-10-S1-A27

**Published:** 2014-03-03

**Authors:** Natasha Gattey, Bahar Bahrani, Peter Hull

**Affiliations:** 1 Department of Medicine, University of Saskatchewan, Saskatoon, Saskatchewan, Canada, S7N 0W8

## Background

Chronic spontaneous urticaria (CSU) is defined as urticaria persisting for more than 6 weeks. An autoimmune basis is held responsible for more than half the cases. In this questionnaire study, we examined responses to treatment, current urticarial activity, effect on lifestyle, frequency of hospital visits, beliefs of causation, and satisfaction with treatment method.

## Methods

The patients were ascertained from the patients seen in the Division of Dermatology at the University of Saskatchewan. 173 patients with CSU had been seen between 2003 and 2013 and an autologous serum skin test (ASST) had been performed on 138 patients. 101 participants responded.

## Results

In the original cohort there were significantly more females than males (130:43). The ASST was positive in 58 patients (42.02%), and only a quarter of these were men (M: F; 12:46).

Of the respondents, 80 were women and 21 men. The age range was 1 year to 81. The mean age was 36 years. The average duration of symptoms was 9.3 years. They included 40 patients who were ASST positive (M: F; 8:32) and 49 negative M:F; 12:37 ). 50 participants no longer had hives. Patients reported being most bothered by pruritus, disturbed sleep, anxiety and their physical appearance including facial swelling. Many (71.2%) had missed work or school because of the urticaria. Almost 22% of participants attributed stress to be a major cause of their CSU.

Twenty-nine patients found antihistamines alone gave adequate relief of urticaria. Prednisone, as prescribed by emergency room physicians and family practitioners, was added as treatment in about a fifth of all the participants.

Twenty ASST positive patients with severe uncontrollable hives were treated with intravenous immunoglobulin (IVIG) available to the patients at no cost through Canadian Blood Transfusion Services. 85.0% of these patients had improved quality of life, with 13 of these patients were now free of urticaria and no longer receiving IVIG. Three patients who did not benefit from IVIG did respond to methotrexate. None of the ASST negative patients received IVIG.

## Conclusion

In this follow-up questionnaire study, about 30% of patients found antihistamines gave effective control. Half the patients had been free of urticaria for at least 3 months. About 40% of patients with CSU had an autoimmune basis as assessed by the ASST and IVIG was a highly effective treatment for this group.

